# Ceramides and Ceramide Scores: Clinical Applications for Cardiometabolic Risk Stratification

**DOI:** 10.3389/fendo.2020.570628

**Published:** 2020-09-29

**Authors:** Mika Hilvo, Vlad C. Vasile, Leslie J. Donato, Reini Hurme, Reijo Laaksonen

**Affiliations:** ^1^Zora Biosciences Oy, Espoo, Finland; ^2^Department of Laboratory Medicine and Pathology and Department of Cardiovascular Diseases Mayo Clinic College of Medicine, Rochester, MN, United States; ^3^Finnish Cardiovascular Research Center Tampere, Tampere University, Tampere, Finland

**Keywords:** cardiovascular disease, coronary heart disease, stroke, heart failure, diabetes mellitus, ceramide, ceramide score

## Abstract

Ceramides are bioactive lipids that have an important role in many cellular functions such as apoptosis and inflammation. During the past decade emerging clinical data have shown that ceramides are not only of great biochemical interest but may also have diagnostic utility. Ceramides have shown independent predictive value for negative cardiovascular outcomes as well as for the onset of type 2 diabetes. Based on abundant published data, risk score using the concentrations of circulating ceramides have been developed and adapted for routine clinical practice. Currently serum ceramides are used clinically as efficient risk stratifiers for primary and secondary prevention of atherosclerotic cardiovascular disease (CVD). A direct cause-effect relationship between CVD and ceramide has not been established to date. As ceramide-specific medications are being developed, conventional strategies such as lipid lowering agents and lifestyle interventions can be used to reduce overall risk. Ceramides can identify a very high-risk coronary heart disease category of patients in need for more intense medical attention, specifically those patients at higher risk as highlighted in the 2019 European Society of Cardiology guidelines for stable chronic coronary syndrome patients. In addition, the ceramide risk score may be used as a decision-making tool in primary prevention patients with moderate CVD risk. Finally, the ceramide risk score may have a unique utility as a motivational tool to increase patient's adherence to medical therapy and lifestyle changes.

## Introduction

Ceramides are bioactive lipids that play important roles in many central processes of human cells, such as apoptosis and inflammation [for additional information see e.g., the following reviews (1–5)]. During the past decade many investigations have revealed a strong association between ceramides and cardiometabolic conditions. An increasing number of studies have shown a link between the development of diabetes and ceramide lipids (6–8). More recently, distinct serum ceramides have shown to be elevated in diabetes (9, 10), and some specific ceramides may predict incident diabetes mellitus type 2 (DM2) even years before the onset of the disease (11). Serum ceramide concentrations have been shown to predict cardiovascular atherosclerotic disease (CVD) such as coronary artery disease (CAD), stroke, as well as heart failure and atrial fibrillation (12–18). These associations were initially observed in small case-control studies or in investigations comparing healthy controls to patients with stable CHD or ACS (12, 19). These promising findings led to a series of larger cohort studies and randomized clinical trials. The initial focus was on CHD but this was later expanded to other cardiovascular (CV) conditions.

The data obtained from these various studies has helped to identify the clinical utility for clinical testing of ceramides because serum ceramide measurements may provide independent and added-value to routinely used diagnostic and prognostic CVD tools. Several studies revealed that ceramides may provide important clinical value, yet the significance of individual ceramides and their mechanistic contribution to the disease pathogenesis has remained uncertain. This has hindered the development of clinical tests of single ceramides. Moreover, a panel test that includes the concentrations of several ceramide concentrations is likely cumbersome to apply to clinical decision making and is not an appealing diagnostic reporting option. To address these shortcomings ceramide-based clinical scores have been developed to aid in end-user comprehension and clinical adoption. The purpose of this review is to provide a summary of CVD- and diabetes-related ceramide data published as of spring 2020 and to depict how the results have been translated into clinical practice.

## Ceramides in Cardiovascular Diseases

### 1) Ceramides in Coronary Heart Disease (CHD)

**Figure 1:**
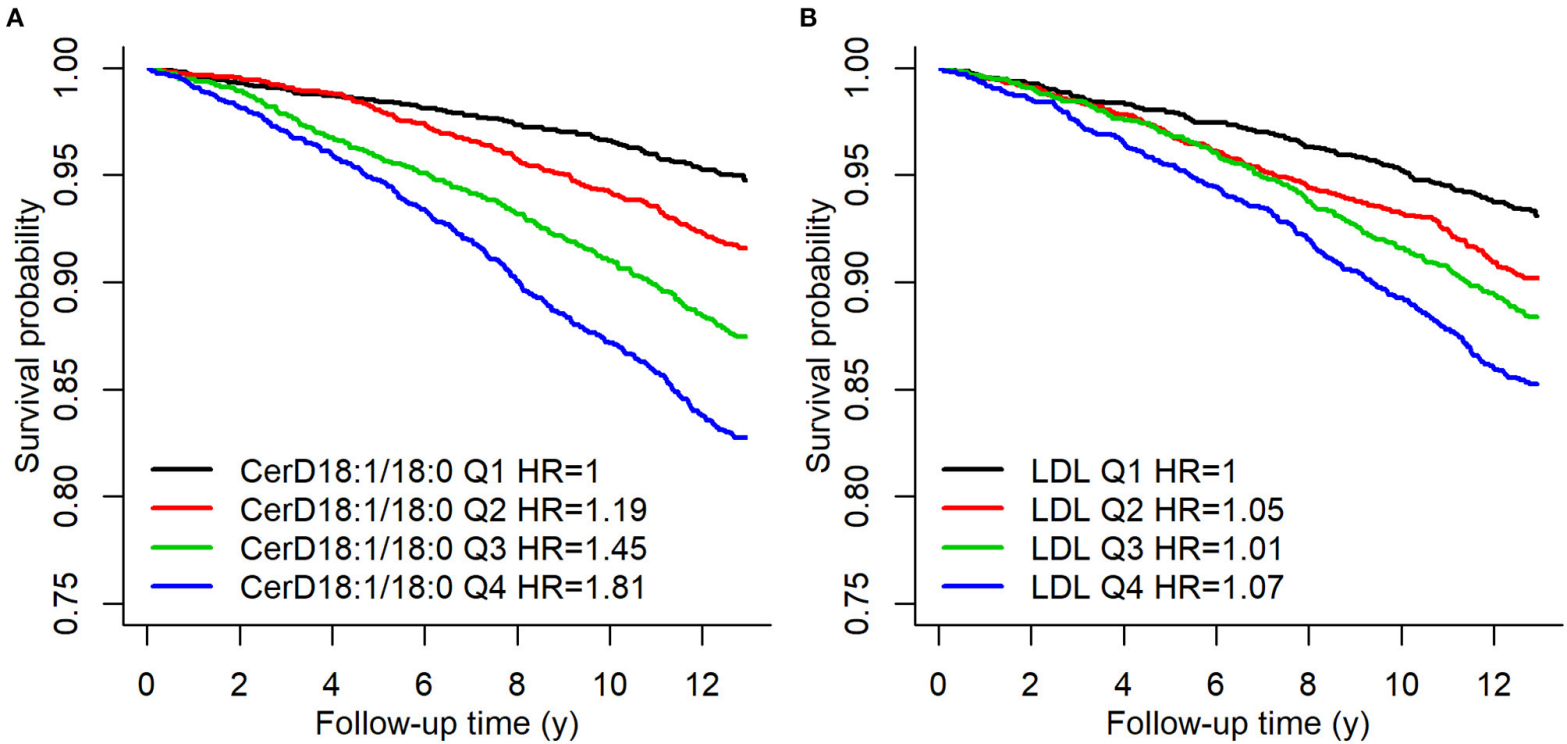
Kaplan–Meier estimates of incident MACE Cer(d18:1/18:0) and LDL-C quartiles. A hazard ratio in the legend indicates the quartile specific hazard relative to the 1st quartile (models stratified for sex). Figure from Havulinna et al. (21). Figure reproduced under the terms of the Creative Commons Attribution License.

The association between ceramides and CHD has been shown in a number of case-control and cohort studies as well as clinical trials (12, 13, 19–23). Cer(d18:1/16:0), Cer(d18:1/18:0), Cer(d18:1/20:0), and Cer(d18:1/24:1), in addition to their ratios with Cer(d18:1/24:0) have been shown to predict the risk of myocardial infarcts (MIs) and CV death in apparently disease free subjects (21) as well as in patients with stable CHD or in secondary prevention after MIs (13). Figure 1 shows Kaplan–Meier estimates of incident major adverse cardiovascular events (MACE) defined as a composite of CHD, ischemic stroke and heart failure (HF) for Cer(d18:1/18:0) and LDL-cholesterol quartiles.

The associations of ceramides with CVD appear stronger for fatal outcomes than non-fatal MIs (13–15, 21). MIs can be precipitated by a number of causes in addition to the traditional model of atherosclerotic plaque rupture (Type 1 MI). Type 2 MIs result from an increased demand or a decreased supply of oxygen which can occur in many conditions such as coronary endothelial dysfunction, coronary artery spasm, coronary artery embolus, tachyarryhthmias/bradyarrhythmias, anemia, respiratory failure, hypertension, or hypotension (24). High sensitive troponin assays can detect even minor ischemic changes seen in non ST-elevation MI or unstable angina. Thus, the phenotypic heterogeneity of MIs may dilute somewhat the prognostic performance of ceramides, although some authors argue that these lipids can provide more specific information to particular MI categories (13). Interestingly, two independent imaging studies have confirmed the localization and association of ceramides with the thin fibrotic plaques with necrotic core, supporting further the assumption that ceramides play an important role in plaque vulnerability in atherosclerotic CAD and thus may associate with fatal complications related to rupture prone inflammatory plaques (25, 26).

**Figure 2:**
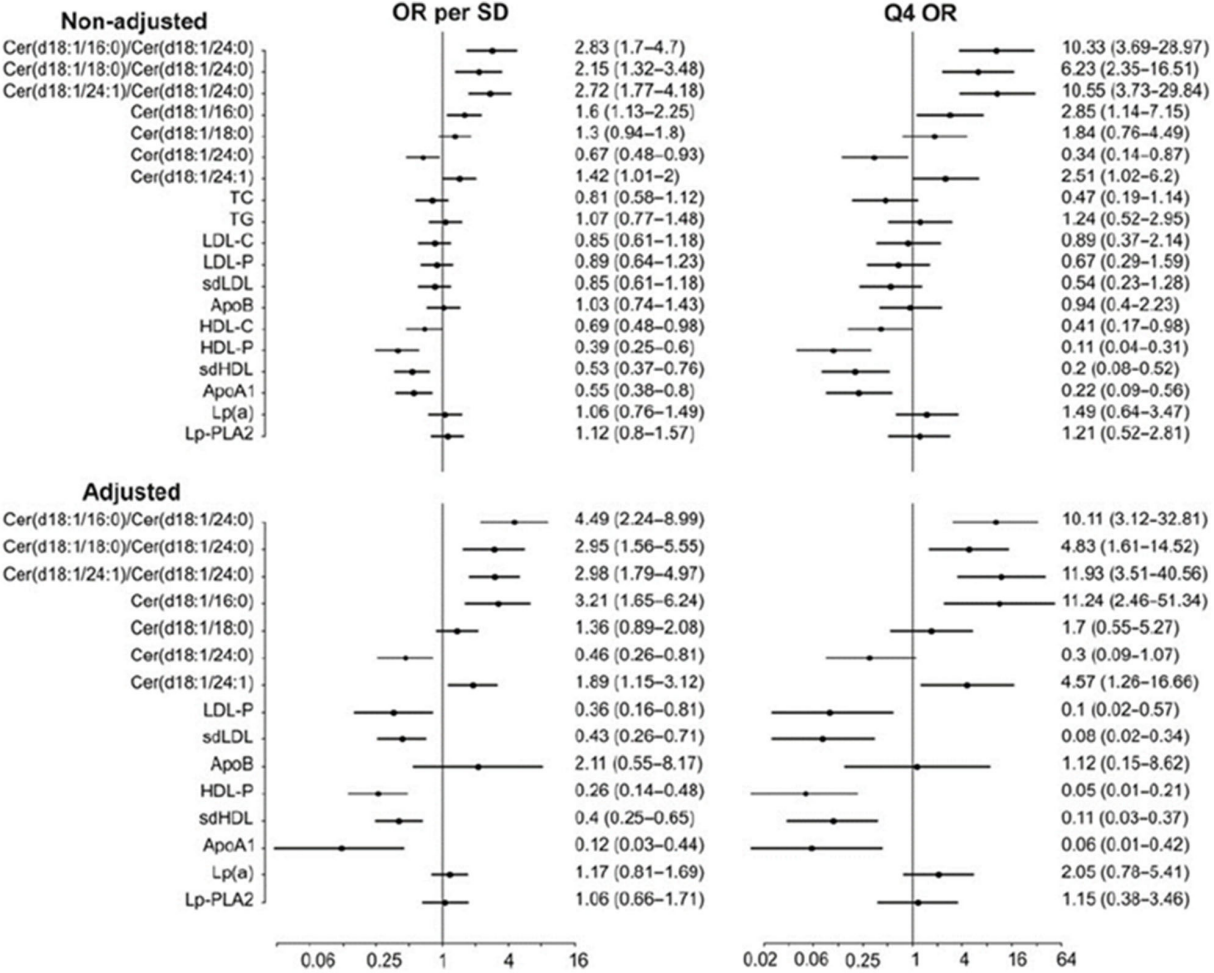
CV death odds ratios for per standard deviation and 4th quartile for different lipid markers and ceramides in the COROGENE study. Adjustment is made for TC, TG, LDL-C, HDL-C, and CRP. Figure from Laaksonen et al. (13). Figure reproduced under the terms of the Creative Commons Attribution License.

Importantly, distinct ceramide ratios improve risk stratification in patients with known CHD as compared to single ceramides or traditional lipid biomarkers. This comparison is illustrated in Figure 2 which shows odds ratios for different ceramides and ceramide ratios as well as traditional lipid parameters in a cohort of stable CHD patients (13). Interestingly, Cer(d18:1/24:1)/Cer(d18:1/24:0) ratio appears consistently associated with CHD risk across studies. This is an intriguing finding since only one double-bond is separating Cer(d18:1/24:1) from Cer(d18:1/24:0) which are otherwise structurally identical. The etiology for the strong CVD risk association of this ceramide ratio still remains obscure.

### 2) Ceramides in Stroke

So far only a few reports have been published regarding ceramides and stroke. Gui et al. evaluated whether ceramides associate with ischemic stroke risk and clinical severity (16). They analyzed concentrations of plasma Cer(d18:1/16:0), Cer(d18:1/22:0), and Cer(d18:1/24:0), in 202 patients with acute ischemic stroke and 202 controls matched for age and sex. Plasma levels of all measured ceramides in stroke patients were significantly higher than in controls (*P* < 0.001). After adjustment for other risk factors, Cer(d18:1/16:0), Cer(d18:1/22:0), and Cer(d18:1/24:0) were associated with higher risk of ischemic stroke [odds ratios for one IQR increase: 2.15 (1.42–2.99); 2.90 (2.13–4.01), and 1.29 (1.10–1.69), respectively] (16). In patients with a minor stroke (*n* = 103), ceramide concentrations were lower than those observed in patients with moderate-to-high clinical severity (*P* < 0.001). Thus, the authors concluded that elevated plasma ceramide levels are predictors of both risk and severity at admission in ischemic stroke patients (16).

Fiedorowicz et al. assessed ceramide and sphingosine-1-phosphate (Sph-1-P) serum concentrations in patients with acute ischaemic stroke, transient ischemic attack (TIA), and age-matched neurological patients without cerebral ischaemia. They recognized two ratios, Sph-1-P/Cer(d18:1/24:1), and Cer(d18:1/24:0)/Cer(d18:1/24:1), with a solid diagnostic potential in ischaemic stroke. They furthermore found Sph-1-P/Cer(d18:1/24:1) ratio to be possibly useful in TIA diagnostics (17).

Based on these studies the relative strength of association between ceramides and stroke seems to be weaker than that of fatal CHD events. More dedicated studies on different stroke phenotypes are warranted since currently it is not entirely clear whether the strength of association is similar for atherosclerotic, thromboembolic and hemorrhagic strokes.

### 3) Ceramides in Heart Failure

Lemaitre et al. have recently reported associations of plasma ceramide and sphingomyelin species with incident heart failure in the Cardiovascular Health Study (18). They examined eight components: ceramides and sphingomyelins that contain palmitic acid (Cer-16:0 and SM-16:0), arachidic acid (Cer-20:0 and SM-20:0), behenic acid (Cer-22:0 and SM-22:0), or lignoceric acid (Cer-24:0 and SM-24:0) in a study with a median follow-up of 9.4 years, where 1,179 cases of incident heart failure were reported among 4,249 study participants. In Cox regression analyses adjusted for risk factors, higher levels of Cer(d18:1/16:0) associated with higher risk of incident heart failure [hazard ratio for one SD increase:1.25 (95% CI, 1.16–1.36)]. In contrast, higher levels of Cer(d18:1/22:0) were associated with lower risk of heart failure in multivariable analyses further adjusted for Cer(d18:1/16:0) [hazard ratio, 0.85 (0.78–0.92)]. Therefore, this study identifies specific lipidomic biomarkers useful for determining risk of incident heart failure.

### 4) Ceramides in Atrial Fibrillation

Ceramides exhibit multiple biological activities that may influence the pathophysiological characteristics of atrial fibrillation (AF) among other CVDs. Jenssen et al. evaluated whether the length of the saturated fatty acid carried by ceramide or their sphingomyelin precursors were associated with incident AF risk among 4,206 Cardiovascular Health Study participants (mean age, 76 years; 40% men). They identified 1,198 incident AF cases over a median 8.7 years of follow-up. In adjusted Cox regression analyses, ceramides with very-long-chain saturated fatty acids were associated with reduced AF risk. In contrast, Cer(d18:1/16:0) was associated with increased AF risk with a hazard ratio of 1.31 (95% CI 1.03–1.66). Their findings suggest that several ceramides are associated with incident AF, and that these associations differ depending on the fatty acid. Ceramides with palmitic acid were associated with increased AF risk, whereas ceramides with very-long-chain saturated fatty acids were associated with reduced AF risk. The associations appeared to be independent of other risk factors and did not differ by subgroups such as age, sex, race, BMI, or prevalent coronary heart disease. Additional studies are needed to evaluate mechanistic linkages between ceramides and atrial fibrillation.

### 5) Ceramides in DM2

The mechanistic role of ceramides in insulin resistance has been reviewed recently (8, 27). Ceramide precursor molecules, dihydroceramides, are elevated years before the onset of type 2 diabetes mellitus (DM2) (28). Additionally, the ceramide ratio Cer(d18:1/18:0)/Cer(d18:1/16:0) predicts the onset of incident DM2 (11). The HR per standard deviation of this ceramide ratio in the population-based FINRISK 2002 study was 2.23 (95% CI 2.05–2.42) and the result remained significant even after adjusting for several traditional risk factors, including BMI, fasting glucose and HbA_1c_. This finding was also validated in stable CAD patients in the WECAC cohort, where the HR was 1.81 (95% CI 1.53–2.14) (11). Thus, this ceramide ratio serves as a strong prognostic marker for the onset of incident DM2.

Taken together, clinical studies have consistently shown a direct and significant association between certain ceramide species and cardiometabolic outcomes. More data are needed to better understand the effect of the genetic contribution and different environmental factors on the circulating ceramide concentrations. In animal models, ceramide inhibitors have been shown to reduce atherosclerotic plaque formation, but studies in humans are currently lacking. Nevertheless, ceramides are clinically useful biomarkers aiding in clinical decision making in patients suspected of atherosclerosis. Different ceramide-based scores, described below, have been developed to help clinical implementation of ceramide-based risk assessment.

## Factors Affecting Circulating Ceramide Concentrations

Serum ceramide concentrations are influenced by certain drugs and lifestyle modifications, including dietary changes and exercise. As ceramides are found in circulating lipoprotein particles, such as LDL-C or HDL-C, their serum levels can be lowered with cholesterol lowering measures (29). It is not surprising that statins, ezetimibe, and PCSK9 inhibitors may lower serum ceramides (12, 29, 30). Furthermore, it has been reported that fenofibrate lowers ceramide concentrations significantly. Croyal et al. conducted analyses of samples collected from 102 patients with type 2 diabetes, enrolled in the FIELD trial, before and after fenofibrate treatment (200 mg/day) (31). They observed a significant decrease in plasma ceramides after fenofibrate treatment, independent of the lipid profile components.

Many studies have shown that metformin has potential to significantly lower ceramide levels in animals. Zabielski et al. concluded that the insulin-sensitizing action of metformin in skeletal muscle is associated with decreased 18-carbon acyl-chain-derived bioactive lipids including Cer(d18:0/18:0) and Cer(d18:1/18:0) in insulin-resistant muscle (32). The same group has also reported that high fat diet augmented the content and fractional synthesis rate (FSR) of diacylglycerol (DAG) and ceramides in the liver which was accompanied by systemic insulin resistance and inhibition of hepatic insulin signaling pathway under insulin stimulation (33). Metformin improved systemic insulin resistance and increased the hepatic insulin signaling cascade and it lowered both the concentration and FSR of ceramides, DAG, and augmented acylcarnitine content and the expression of mitochondrial markers. The authors suggest that in the liver, the insulin sensitizing effect of metformin depends on increase of mitochondrial β-oxidation, which defends from hepatic buildup of both the ceramides and DAGs and preserves insulin sensitivity under HFD consumption (33). Recently, Marfella et al. observed that pathogenesis of human diabetic cardiomyopathy started with cardiomyocyte lipid accumulation following heart transplantation in diabetes mellitus recipients and that metformin use was associated with reduced ceramide and triglyceride accumulation independent of immunosuppressive therapy (34). Furthermore, insulin sensitizing thiazolidinediones have been found in mouse models to reduce ceramides in skeletal muscle (35). Later, Warshauer et al. observed in a single-center, randomized, double-blind, placebo-controlled trial comparing pioglitazone to placebo that pioglitazone in individuals with metabolic syndrome induced a potent decrease in plasma ceramides, and some of the changes correlated with changes in insulin resistance and adiponectin levels (36). Taken together, it appears that metformin and pioglitazone in addition to lipid lowering compounds have a significant potential to alter ceramide metabolism in ways that might be clinically beneficial. Recently Sodium-glucose cotransporter-2 (SGLT2) inhibitors have shown to reduce CV events in DM2 and HF patients (37, 38), but the possible linkage between ceramides and SGLT2 treatments remains to be evaluated.

Diet may also affect ceramide levels. Luukkonen et al. have shown that saturated fat intake induce insulin resistance and endotoxemia in addition to increasing multiple plasma ceramides in overweight subjects fed with an additional 1,000 kcal/day of saturated fat (39). Additionally, certain dietary interventions may lower circulating ceramide levels. Mathews et al. assessed the efficacy of a fruit and vegetable intervention on overall metabolic health, circulating ceramide supply and inflammatory status in young adults (40). They observed in this pilot study of 36 young adults participating in the 8-week free-living nutritional intervention that a short-term nutritional intervention can lower serum ceramide concentrations. Future studies with larger sample sizes is needed to better understand the effects of nutrients on distinct ceramide concentrations.

Interestingly, in the PREDIMED trial Wang et al. reported a positive association between baseline plasma ceramide concentrations and incident CVD. Importantly, they showed that a Mediterranean dietary intervention may alleviate potential harmful effects of raised plasma ceramide concentrations on CVD (41). Their study population consisted of 980 participants from the PREDIMED trial (Prevención con Dieta Mediterránea), including 230 incident cases of CVD and 787 randomly selected participants at baseline (including 37 overlapping cases) followed for ≤7.4 years. The participants were randomized to a Mediterranean diet supplemented with extra virgin olive oil, a Mediterranean diet supplemented with nuts, or a control diet. The traditional Mediterranean diet enriched with extra virgin olive oil or nuts showed the potential to alleviate the harmful effects of high ceramide concentrations on CVD outcomes. However, further studies are needed to replicate these intriguing results in other populations as well as to investigate potential mechanisms.

Reidy et al. have recently reviewed the relationship between skeletal muscle ceramides and insulin resistance in response to increased physical activity (42). Their review of the literature indicated that chronic exercise reduces ceramides in individuals with obesity, diabetes or hyperlipidemia. However, in metabolically healthy individuals increased physical activity can improve insulin sensitivity independent of changes in skeletal muscle ceramide content (42). Kasumov et al. studied 24 adults with obesity and normal glucose tolerance (NGT, *n* = 14) or diabetes (*n* = 10) before and after a 12-week supervised exercise-training program (5 days/week, 1 h/day, 80–85% of maximum heart rate) (43). Concentrations of Ceramides were similar in subjects with obesity and NGT and in subjects with diabetes, despite differences in glucose tolerance. Notably, exercise significantly reduced plasma concentrations of Cer(d18:1/14:0), Cer(d18:1/16:0), Cer(d18:1/18:1), and Cer(d18:1/24:0) in all subjects following the intervention (*P* < 0.05). Petrocelli et al. performed a reversed exercise study by evaluating the effect of bed rest on circulating ceramides (44). As acute bed rest places older adults at risk for health complications by disrupting homeostasis in many organ systems including the cardiovascular system, they hypothesized that circulating ceramides predictive of poor cardiometabolic outcomes would increase following 5-days of bed rest. In their study, 35 healthy younger and older men and women (young: *n* = 13, old: *n* = 22) underwent 5 days of controlled bed rest. The primary observations were that circulating ceramides decreased while ceramide ratios and the (CERT1) score (see below CERT1 score), associated with CV risk, increased primarily in older adults. It is noteworthy, that these findings were independent of changes in circulating lipoprotein levels (44).

In summary, ceramides are modifiable by certain drugs, lifestyle, and environmental factors. The significance of genetic factors affecting serum ceramide concentrations and ceramide metabolism at a cellular level still remain to be investigated. While serum ceramides are novel biomarkers of CVD, their potential causality has not yet been established. This would be important for promoting attempts to develop ceramide-targeted drugs. Initial data from animal experiments suggest that ceramide synthetic pathways may offer targets for drug development as inhibition of serine palmitoyltransferase (SPT) has shown to significantly reduce the development of atherosclerosis in a murine model (45–47); similarly dihydroceramide desaturase 1 (DES1) inhibition has proven to be beneficial in treatment of diabetes mellitus in mice (48).

## Ceramide Score as the Clinical Solution

### CERT1 Score

**Figure 3:**
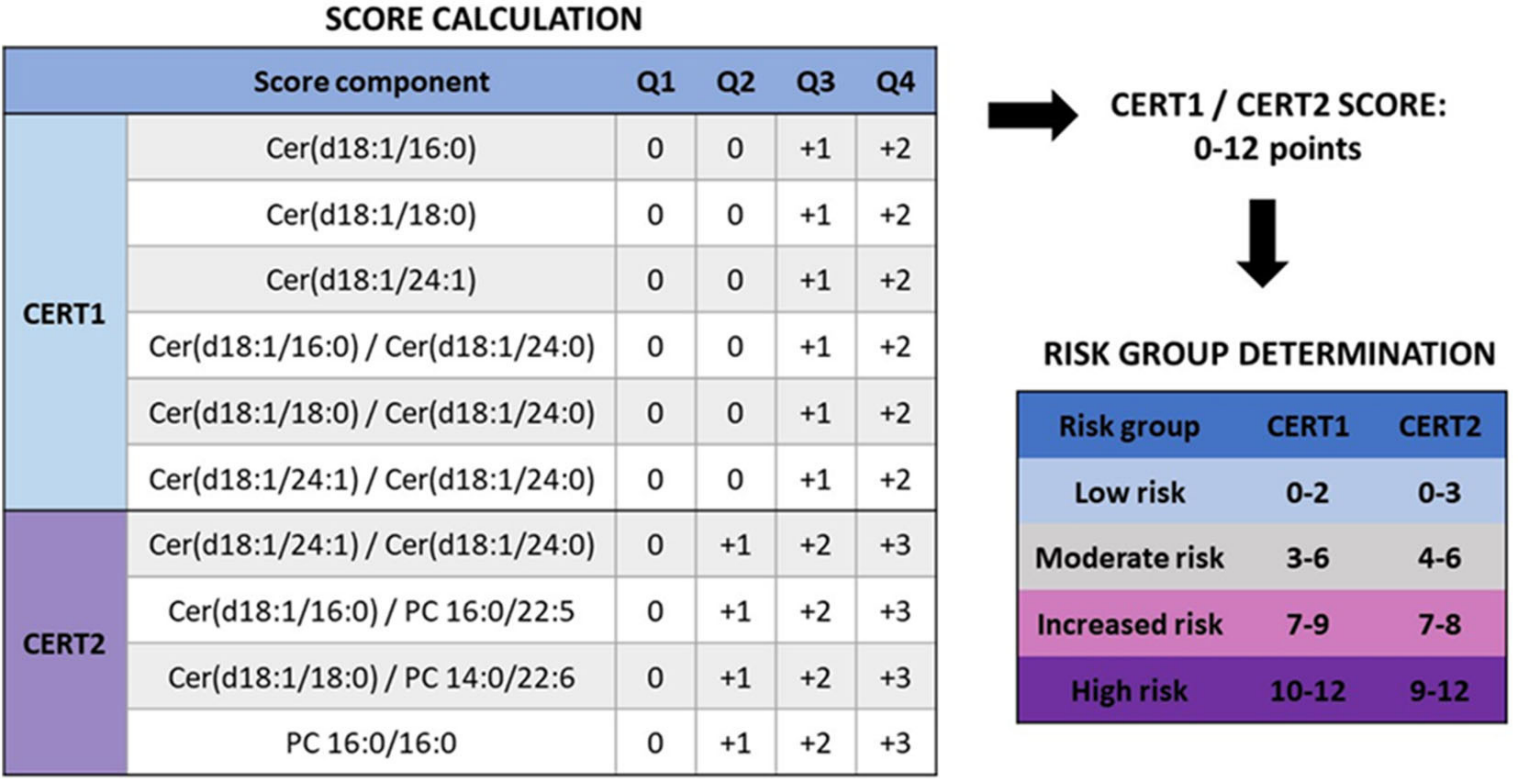
Calculation of CERT2 risk scores and determination of CVD risk groups (14). When determining the score for a subject, it is evaluated to which quartile the person belongs with regards to each score component. CERT1 has six components, and only quartiles 3 and 4 give points, while CERT2 has only 4 components but already the second quartile yields a risk point. Thus, the scale for both of these scores is the same, 0–12 points. Based on these the subject is categorized to one of the CVD risk categories. Figure reproduced under the terms of the Creative Commons Attribution License.

Since panels of single ceramide species or other lipid combinations are rather cumbersome for clinical practice, CERT1 risk score was developed for clinical use based on ceramide concentrations and their ratios (13, 21). To calculate the CERT1 score, Cer(d18:1/16:0), Cer(d18:1/18:0), and Cer(d18:1/24:1) concentrations and their ratios to Cer(d18:1/24:0) are determined. The scoring system assigns 2 points to those with concentrations or ratios in the fourth quartile, 1 point to values in the third quartile, and 0 points to the first and second quartiles, with total CERT1 scores ranging from 0 to 12 (Figure 3).

Based on CERT1, patients are stratified into four risk categories (low–moderate–increased–high) (Figure 3) and a linear CVD risk increase is observed along with the increasing score both in patients with a stable CHD and acute coronary syndrome (ACS) (Table 1). When comparing the high to low risk category, there is a 4.2- and 6.0-fold relative risk increase in patients with stable CHD and ACS, respectively. When subjects were sorted according to their LDL-C concentrations and divided in quartiles, the high-risk patient quartile was not identified using only the LDL-C concentration, supporting the view that the ceramide score could improve risk stratification beyond LDL-C (13). The performance of the test was further validated in a large-scale population-based study (FINRISK 2002; *N* = 8101). In this cohort a 4.2-fold relative risk increase was observed when comparing the high to low risk category (21) (Table 1).

**Table 1:** CERT1 and risk of CV death in primary prevention, stable CAD patients and ACS patients.

	**Primary prevention**			**Stable CAD**			**ACS**		
	**Category**	**Risk (%)^a^**	**Relative risk**	**Category**	**Risk (%)^b^**	**Relative risk**	**Category**	**Risk (%)^c^**	**Relative risk**
CERT1	0–2	1.2%	Ref.	0–2	2.7%	Ref.	0–2	1.6%	Ref.
	3–6	1.9%	1.6	3–6	4.8%	1.8	3–6	2.6%	1.7
	7–9	3.8%	3.2	7–9	6.9%	2.5	7–9	3.3%	2.1
	10–12	5.1%	4.3	10–12	11.4%	4.2	10–12	9.4%	6.0
LDL-C (mmol/L)	≤ 2.9	1.8%	Ref.	≤ 2.6	6.6%	Ref.	≤ 2.7	4.8%	Ref.
	2.9–3.8	2.2%	1.2	2.6–3.7	4.8%	0.7	2.7–3.7	2.9%	0.6
	3.8–4.7	2.8%	1.5	3.7–4.5	3.5%	0.5	3.7–4.5	1.1%	0.2
	≥4.7	3.4%	1.8	≥4.5	4.1%	0.6	≥4.5	1.1%	0.2

*LDL-C was divided into groups in the same proportion as CERT1. Data from (13, 21)*.

a *13-year risk*,

b *5-year risk*,

c *1-year risk*.

### CERT2 Score

As phosphatidylcholines (PCs) have shown prognostic value for CV events (49), it was investigated whether the ceramide test score (CERT1) could be upgraded by adding distinct PCs to the score. Main drivers of the lipid species choice were analytical stability, ability to incorporate into the existing CERT1 assay, and statistical strength across a number of clinical cohorts. The novel ceramide test score, named CERT2, was developed in the WECAC study and validated in the LIPID and KAROLA studies, by selecting the test components in a stepwise manner (14). The original CERT1 score contained three single ceramides and three ceramide/ceramide ratios, whereas the CERT2 score had one ceramide/ceramide ratio, two ceramide/PC ratios and a single PC. Ceramide-PC ratio components of the CERT2 test showed higher HRs than the previously published ceramide–ceramide ratios, across all three studies (14).

**Figure 4:**
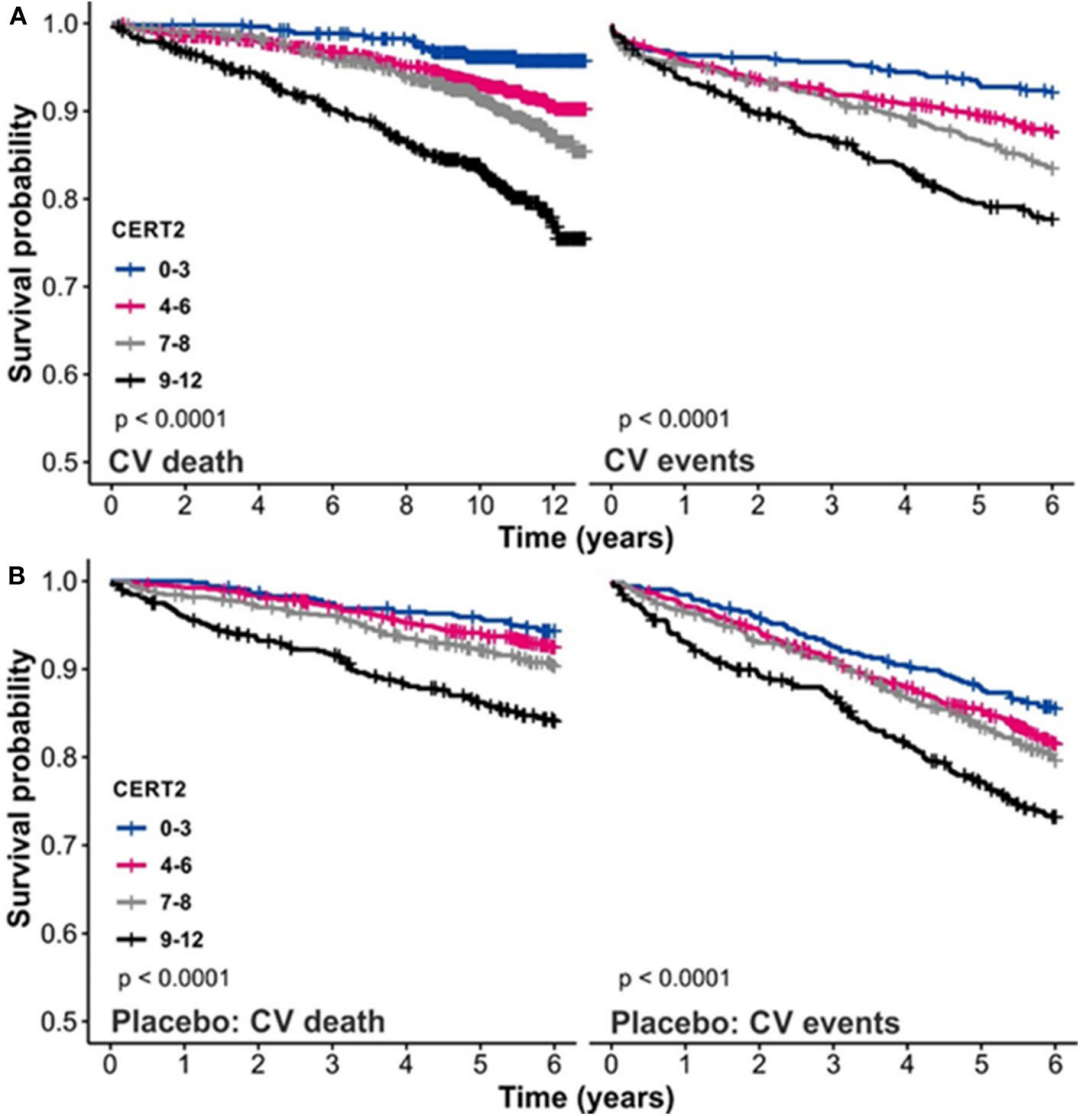
Kaplan–Meier curves for CERT2 **(A)** in the WECAC cohort and **(B)** in the placebo treatment arm in the LIPID trial. Figure adapted from Hilvo et al. (14). Figure reproduced under the terms of the Creative Commons Attribution License.

**Figure 5:**
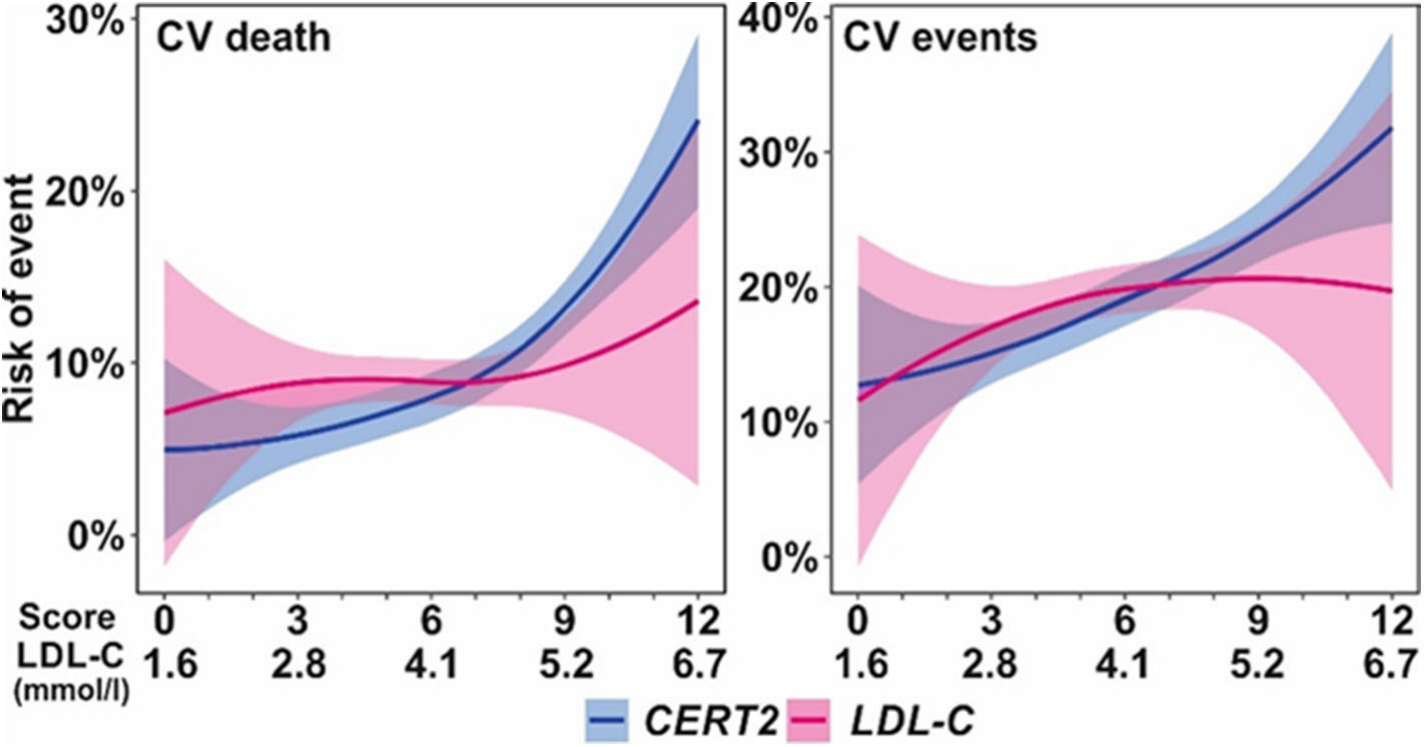
Risk (6 years) curves for CERT2 and LDL-C in the LIPID trial placebo treatment arm. Figure adapted from Hilvo et al. (14). Figure reproduced under the terms of the Creative Commons Attribution License.

The CERT2 risk estimation tool showed improved performance metrics and can be used to reliably stratify CHD patients for their risk of CV events, especially CV death (14). The performance of the CERT2 score, in addition to other CV biomarkers, was evaluated in three independent large cohorts of CHD patients. For CV death, the HRs (95% confidence interval) per standard deviation (SD) for CERT2 were 1.50 (1.35–1.68) in WECAC, 1.51 (1.38–1.65) in the LIPID trial, and 1.62 (1.32–2.00) in KAROLA. For all the investigated biomarkers, the HRs for CV events were lower than for CV death (14). Performance comparison of CERT2, CERT1, and other CVD biomarkers are provided in Table 2. This comparison shows that HR values of CERT2 exceed those of CERT1 and other markers, including the routinely used lipid parameters and high sensitivity C-reactive protein (hsCRP). Furthermore, in all three studies the risk for CV death and CV events increased along with increasing CERT2 score and risk group. For CV death, a 3.5- to 5.4-fold increase in risk was observed in different cohorts between the highest and lowest risk groups for CERT2, while the risk increase was more modest for CERT1 and for LDL-C which is widely used in clinical risk estimation assessments (14) (Table 3). One of the major advantages of this study was the inclusion of samples obtained from patients randomized to the placebo arm of the LIPID trial. This avoids confounding effects caused by lipid lowering treatments. Importantly, the result obtained in the statin free population of the LIPID trial confirmed previous observations from the WECAC study and demonstrated the value CERT2 to assess risk in CHD (Figure 4). Figure 5 shows the risk curves both for CERT2 and LDL-C in the placebo-treated patients in the LIPID trial. The risk curve for CERT2 increases linearly while the risk curve for LDL-C showed a slight risk increase only for the highest LDL-C levels > 5.2 mmol/l (200 mg/dL).

**Table 2:** Hazard ratios (HR) per standard deviation for the CERT scores predicting cardiovascular death and events, and comparison with other cardiovascular biomarkers in the WECAC cohort.

	**CV death**				**CV events**			
**Variable**	**HR (95% CI)^a^**	***p*-value**	**HR (95% CI)^b^**	***p*-value**	**HR (95% CI)^a^**	***p*-value**	**HR (95% CI)^b^**	***p*-value**
CERT2	1.50 (1.35, 1.68)	2.6E-13	1.44 (1.28, 1.63)	3.1E-09	1.36 (1.25, 1.48)	5.0E-12	1.29 (1.17, 1.42)	1.7E-07
CERT2-TnT	1.79 (1.59, 2.00)	<2.2E-16	1.63 (1.44, 1.85)	2.2E-14	1.53 (1.40, 1.68)	<2.2E-16	1.39 (1.26, 1.54)	7.9E-11
CERT1	1.27 (1.14, 1.41)	7.9E-06	1.23 (1.09, 1.38)	5.4E-04	1.24 (1.14, 1.35)	8.5E-07	1.18 (1.08, 1.30)	4.1E-04
LDL-C	1.05 (0.94, 1.17)	n.s.	1.15 (1.01, 1.30)	0.032	1.03 (0.94, 1.12)	n.s.	1.13 (1.02, 1.24)	0.015
HDL-C	0.81 (0.72, 0.91)	3.8E-04	0.95 (0.84, 1.07)	n.s.	0.83 (0.75, 0.91)	8.8E-05	0.94 (0.85, 1.04)	n.s.
TG	1.15 (1.04, 1.27)	0.005	1.07 (0.96, 1.19)	n.s.	1.15 (1.07, 1.24)	2.8E-04	1.08 (1.00, 1.18)	n.s.
ApoB	1.15 (1.03, 1.28)	0.009	1.33 (1.03, 1.72)	0.031	1.09 (1.00, 1.18)	n.s.	0.99 (0.80, 1.23)	n.s.
ApoA1	0.79 (0.71, 0.89)	8.6E-05	0.91 (0.74, 1.12)	n.s.	0.84 (0.76, 0.92)	1.6E-04	0.94 (0.80, 1.10)	n.s.
CRP	1.12 (1.05, 1.20)	0.001	1.10 (1.02, 1.19)	0.010	1.08 (1.02, 1.15)	0.013	1.06 (0.99, 1.14)	n.s.
TnT	1.43 (1.31, 1.55)	2.2E-16	1.30 (1.19, 1.43)	1.7E-08	1.35 (1.26, 1.45)	4.1E-16	1.23 (1.14, 1.34)	1.8E-07
Lpa	1.13 (1.02, 1.25)	0.020	1.13 (1.01, 1.26)	0.029	1.12 (1.03, 1.21)	0.010	1.10 (1.01, 1.19)	0.034
TMAO	1.06 (0.97, 1.16)	n.s.	1.02 (0.94, 1.12)	n.s.	1.04 (0.96, 1.12)	n.s.	1.01 (0.93, 1.10)	n.s.

*n.s., not significant. Data from Hilvo et al. (14)*.

a Age as time scale, stratified by vitamin B intervention;

b *additionally adjusted for sex, statin, diabetes, hypertension, current smoking, previous MI, previous stroke, BMI, LDL-C, HDL-C, TG, CRP*.

*LDL-C, LDL-cholesterol; HDL-C, HDL-cholesterol; TG, triglycerides; ApoB, apolipoprotein B, ApoA1, apolipoprotein A1; hsCRP, high-sensitivity C-reactive protein; TnT, high-sensitivity troponin-T; Lp(a), lipoprotein(a); TMAO, trimethylamine N-oxide; NS, not significant*.

**Table 3:** Risk for CV death in different CERT score groups, and with respect to LDL-C.

	**CERT2**			**CERT1**			**LDL-C**		
	**Group**	**Risk**	**Rel.risk**	**Group**	**Risk**	**Rel.risk**	**mmol/l**	**Risk**	**Rel.risk**
WECAC	0–3	3.5%	Ref.	0–2	5.1%	Ref.	≤ 2.1	7.3%	Ref.
	4–6	6.4%	1.8	3–6	6.3%	1.2	2.1–3.2	7.7%	1.1
	7–8	7.8%	2.2	7–9	11.1%	2.2	3.2–4.2	8.1%	1.1
	9–12	15.1%	4.3	10–12	15.5%	3.0	≥4.2	6.6%	0.9
LIPID	0–3	4.3%	Ref.	0–2	5.9%	Ref.	≤ 3.2	8.8%	Ref.
	4–6	6.3%	1.5	3–6	8.7%	1.5	3.2–4.0	7.9%	0.9
	7–8	9.9%	2.3	7–9	8.9%	1.5	4.0–4.6	7.9%	0.9
	9–12	15.2%	3.5	10–12	14.8%	2.5	≥4.6	9.7%	1.1
KAROLA	0–3	2.6%	Ref.	0–2	3.5%	Ref.	≤ 2.4	7.6%	Ref.
	4–6	5.4%	2.1	3–6	7.0%	2.0	2.4–3.1	6.5%	0.9
	7–8	7.8%	3.0	7–9	11.8%	3.4	3.1–3.8	7.1%	0.9
	9–12	13.8%	5.4	10–12	12.7%	3.7	≥3.8	5.0%	0.7

*For comparison, LDL-C was divided into groups (Q1-Q4) in the same proportion as CERT2. For the WECAC and KAROLA studies, the risk is for 10 years and for LIPID trial 6 years. Data from Hilvo et al. (14)*.

*Ref. = reference category*.

In the large-scale STABILITY trial of optimally treated secondary prevention patients, CERT2 was able to significantly improve HR for CV outcomes in a study population covering various geographical locations worldwide (15). There were differences in the score distribution by location, and further work is needed to delineate the factors behind this observed distribution difference. There were statistically significant associations between CERT2 and all CV outcomes including stroke and heart failure. For CERT2, the highest unadjusted hazard ratios (HRs) per SD were observed for CV death (HR, 1.57; 95% CI, 1.45–1.69), all-cause death (HR, 1.54; 95% CI, 1.45–1.64), and heart failure hospitalization (HR, 1.52; 95% CI, 1.35–1.70). For stroke the unadjusted HR was 1.29 (1.15–1.46).

**Figure 6:**
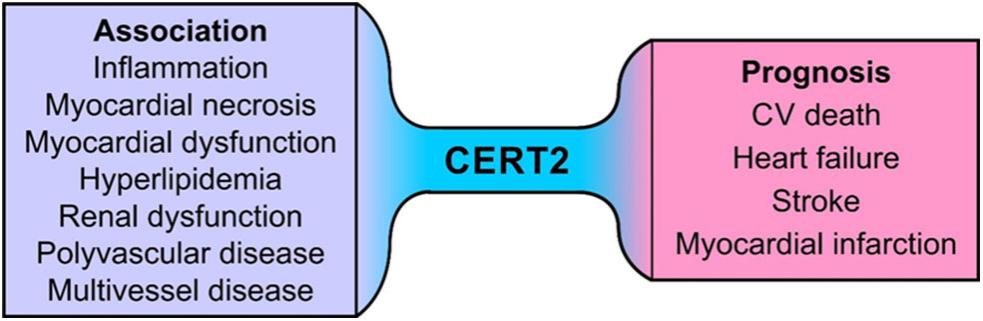
Association of CERT2 with pathophysiological processes and cardiovascular outcomes.

In STABILITY the CERT2 score was associated significantly with smoking and multivascular disease as well as with multivessel CAD. Furthermore, patients with renal dysfunction had higher CERT2 scores, while the association with high blood pressure and DM was much weaker. In addition, CERT2 was significantly related with the levels of lipid biomarkers (LDL-C and triglyceride) and supported the view that ceramides are essential constituents of circulating lipoproteins. The CERT2 score was prognostic even after adjustment for LDL-C and triglyceride levels. This indicates that sphingolipids may be important for cardiovascular disease beyond conventional lipids. The CERT2 score was also significantly associated with inflammatory markers (hs-CRP and IL-6), suggesting that certain ceramide levels might be a surrogate of vascular inflammation. These findings suggest that CERT2 could assess both plaque burden and inflammatory residual risk in patients with stable CHD. Higher CERT2 score was also associated with higher hs-TnT and NT-proBNP concentrations. These findings indicate that alterations in ceramide expression might be associated with, and possibly contribute to, myocardial injury and/or myocardial dysfunction. CERT2 score is a useful tool to determine residual risk in patients with stable CHD as it is associated with all CV events and reflects disturbances of several key mechanisms for CVD such as dyslipidemia, inflammation, myocardial injury, and renal dysfunction (15) (Figure 6).

### Unbiased Machine Learning Based SIC Score

A new approach to identify associations between serum ceramides and coronary artery disease (CAD) was introduced by Poss et al. (50). They performed targeted lipidomics on serum samples from individuals with familial CAD (*n* = 462) and population-based controls (*n* = 212) to study the association between serum sphingolipids and CAD, using unbiased machine learning to find sphingolipids related with CAD. They observed that in this setting nearly every sphingolipid measured (30 out of 32) was significantly elevated in subjects with CAD compared to controls. The authors generated a novel sphingolipid-inclusive CAD risk score, termed SIC, that demarcates patients with CAD independently and more effectively than conventional clinical CVD biomarkers including serum LDL cholesterol and triglycerides (49). This new metric comprises several minor lipids that, according to authors, likely serve as measures of flux through the ceramide biosynthesis pathway rather than the abundant deleterious ceramides that are included in other ceramide-based scores. Despite the fact that this cross-sectional study used case and control samples collected from different sources and time points 10 years apart in 1980s and 1990s, the results suggest that comprehensive sphingolipid panels could also be considered as a measure of CVD. However, these findings need to be further validated in large-scale cohort studies. The use of a large panel of minor ceramides may pose practical issues in analytical validation for clinical use.

### Diabetes Score (dScore)

Compared to the CVD risk scores, a different approach was taken when using the ceramide ratio Cer(d18:1/18:0)/Cer(d18:1/16:0) in a clinically applicable scoring system for predicting the onset of diabetes (dScore). In addition to the lipid ratio, this scoring system incorporates the patient's age, sex and BMI, and gives a 10-year absolute risk (scale 0–100) of developing DM2 (11). The subjects are grouped into risk categories: low risk is defined as <5%, moderate risk 5–15% and high risk more than 15% probability of developing DM2 in the next 10 years. It is noteworthy that the ceramide ratio, and consequently the dScore, was significantly reduced in persons with a weight loss of 5% or more (11), which is consistent with earlier dietary interventions showing that weight loss of a few kilograms is enough to reduce the risk of developing diabetes (51).

## Clinical Use of Scores

The CERT1 ceramide score has been implemented in clinical use in private and public practice both in Finland and at Mayo Clinic in the USA. The score includes a single easy to understand readout for physicians as opposed to a list of multiple ceramide results each with separate reference values. The score calculation is based on ratios of distinct ceramides, which offers another advantage since an occasional variation in concentrations of single ceramides does not significantly affect the ratio. The score-based reporting system also allows an opportunity for graphical visualization of the results in a condensed form which is simple to understand and could be useful to share with patients. Ceramide laboratory measurements are performed by high-throughput robotic enabled mass spectrometry instrumentation. Mass spectrometry is classically associated with high-sensitivity requirements of hormonal measurements, vitamin D or immunosuppressants among other. The technology is highly robust, easy to set up and the quality controlled specific standards conform to all required regulatory standards. The cost and speed of daily operation is comparable to antibody-based assay solutions in high-volume laboratories and the equipment is becoming more routinely available in most high volume clinical laboratories.

The studies provide robust evidence that ceramide scores (CERT1, CERT2) can be used for risk stratification in both primary and secondary prevention settings. These scores allow for instance a rapid identification and stratification of residual risk in patients with known CHD who might benefit from more in-depth scrutiny and perhaps more aggressive therapy. Most risk prediction systems in clinical practice use age as part of the assessment. Age is most likely the most powerful biomarker for risk prognostication in general, but it has also significant limitations as it may delay timely intervention in younger and middle-aged individuals. Biomarkers that are not age-dependent allow an earlier detection of risk and prevention. Conversely, many elderly patients may be at relatively low risk despite their age-driven risk calculator scores. In these cases an age-independent biomarker may be useful as these patients might do well without aggressive interventions.

In the 2019 ESC/EAS Guidelines (52) for the management of dyslipidaemias the selection of intervention strategies depends on the cardiovascular risk (assessed e.g., with SCORE) and theconcentration of untreated low-density lipoprotein cholesterol. In many cases the selection between lifestyle modification or pharmacologic intervention is clear. In subjects at moderate CV risk, consideration of lipid lowering agents is recommended. Clinicians frequently evaluate patients with a moderate CV mortality risk (>1 to <5% in 10 years, assessed with SCORE) and LDL-C concentrations between 2.6 and 4.9 mmol/L (100 and 190 mg/dL) where the decision of initiation of a lipid lowering agent is not obvious or clearly recommended by guidelines. Especially in these subjects, additional risk assessment with CERT could be useful for risk stratification in order to identify the subgroup of patients at higher risk who may benefit from more aggressive intervention. This is also recapitulated in the US guidelines where patients deemed at intermediate risk by the ASCVD risk assessment calculator may be further stratified after the assessment of risk enhancers such as the coronary calcium (CAC) scoring (53). The latter testing, which is extensively used in primary preventive cardiology, has some important limitations as it does not capture information about the regional distribution of calcification within the coronary tree (54). Furthermore, CAC score does not incorporate information on the number, size of calcified coronary lesions or biology of calcification and atherosclerosis (54). Perhaps as a consequence of these limitations, events are observed in patients scored as CAC = 0, and many older patients with high CAC scores never experience events—pointing to potential room for improvement (54). Therefore, a simple blood test such as CERT or CERT2 could be clinically informative in a wide variety of patients.

In the latest ESC guideline for stable chronic coronary syndromes (sCCS) (55) the risk variability is described and emphasis is placed on the “very high risk” patients who carry a more than three percent annual mortality risk. Guidelines recommend the use of additional diagnostic tools to identify these patients at the highest risk. However, the recommended imaging methods may not always be available (e.g., stress echocardiography or MRI) and invasive angiography may not always be warranted. Thus, the identification of patients with CHD at highest risk can be significantly and efficiently improved by the addition of a blood test such as one of the ceramide risk scores. CERT has already been used clinically for secondary prevention both in Finland and the US. A combination of the newly developed CERT2 and high sensitivity troponin T (hsTNT) has already been suggested by Hilvo et al. to drastically improve prognostic performance (14).

Different medical treatments may be prescribed in the future based on specific risks and such treatments may become truly personalized. It has been shown that cholesterol lowering agents such as ezetimibe or PCSK9 inhibitors result in higher absolute risk reductions in patients who have a higher baseline risk (56, 57). Ceramide scores could be used in the selection of CHD patients at greatest risk or in primary prevention patients for novel and patient-tailored individualized treatments. This approach appears appealing for lipid lowering drugs because the primary lipid target, LDL-cholesterol, or its proxy, total cholesterol, have rather limited association with outcome in elderly subjects (58–60) and in patients with established CHD (13, 14), thus for these patients ceramide scores could provide an instrument with added value for more tailored treatments.

Another potential field of interest for risk assessment could be antiplatelet therapy. Dual antiplatelet therapy is recommended and routinely used in secondary prevention after myocardial infarction. The duration of antiplatelet therapy after revascularization remains controversial and the risk of bleeding is of concern. Several factors are taken into consideration when assessing the risk of in-stent thrombosis vs. the risk of bleeding and clinicians often face difficult decisions not described directly in the guidelines when taking care of the complex cardiology patient. Ceramide scores could potentially be used in identifying patients at higher risk who might benefit the most from extended dual anti-thrombotic therapy. Finally, drugs that target ceramides directly may become available for clinical use in addition to drugs that reduce circulating ceramide levels indirectly via HMG-CoA or PCSK9 inhibition. Additionally, lifestyle changes such as weight reduction have shown to reduce the ceramide-based diabetes risk score (11). As the CERT2 score contains also phosphatidylcholines containing omega-3 fatty acids it appears plausible that treatments such as EPA could significantly lower the risk, but this remains to be determined.

Despite rather comprehensive scientific evidence there are still significant barriers limiting more widespread use of ceramides in CVD risk assessment that are typical for any novel diagnostic test. First, the test must be widely available through multiple outlets. Currently, in the US CERT1 is offered only by one reference laboratory. Serving as a pilot for Europe as a whole, the test is available in nearly all private healthcare centers in Finland and has been incorporated into the portfolio of the public health care offering in that country. Second, most clinical outcomes studies using ceramides have been reported within only the last decade so most practicing clinicians are unaware of the clinical utility of ceramide testing. To overcome this challenge, physician education and dissemination of the scientific evidence to various groups of physicians is required. Widespread and effective dissemination is possible only for global marketing organizations and ceramides are lacking such support. Acceptance to ESC/EAS and AHA/ACC guidelines would be another avenue to more widespread clinical uptake. As described earlier in this review, there are certain conditions particularly in the recent ESC 2019 guidelines where we believe ceramide testing could well be justified. Third barrier not only for ceramides but for all new diagnostics is the stringent reimbursement policies of insurance companies as their requirements are often impractical for novel CVD tests and, thus, the established older tests are being reimbursed and used despite limitations of their clinical performance.

## Conclusions

Ceramide scores are currently used in clinical practice to identify residual risk in patients with established CHD and may further be used in primary prevention to identify subjects at elevated risk who might benefit from more intensive prevention. CERT scores can be used for follow-up and to further motivate patients to initiate or continue medical and life-style recommendations.

## Author Contributions

RL wrote the manuscript. MH, VV, LJD, and RH critically revised the manuscript. All authors contributed to the article and approved the submitted version.

## Conflict of Interest

Zora Biosciences Oy holds patent disclosures related for the diagnostic and prognostic use of ceramides and phospholipids in CVD. MH, RH, and RL are employees. RH and RL are shareholders of Zora Biosciences Oy. The remaining authors declare that the research was conducted in the absence of any commercial or financial relationships that could be construed as a potential conflict of interest.
